# Chondroitin Sulfate Expression in Perineuronal Nets After Goldfish Spinal Cord Lesion

**DOI:** 10.3389/fncel.2018.00063

**Published:** 2018-03-12

**Authors:** Akihito Takeda, Masashige Shuto, Kengo Funakoshi

**Affiliations:** ^1^Department of Neuroanatomy, Yokohama City University School of Medicine, Yokohama, Japan; ^2^Yokohama City University School of Medicine, Yokohama, Japan

**Keywords:** teleost, spinal cord injury, perineuronal net, chondroitin sulfate, dextran amine

## Abstract

Perineuronal nets (PNNs) surrounding neuronal cell bodies regulate neuronal plasticity during development, but their roles in regeneration are unclear. In the PNNs, chondroitin sulfate (CS) is assumed to be involved in inhibiting contact formation. Here, we examined CS expression in PNNs in the ventral horn of a goldfish hemisected spinal cord in which descending axons regenerate beyond the lesion to connect with distal spinal neurons. In intact fish, chondroitin sulfate A (CS-A)–positive PNNs accounted for 5.0% of HuC/D-immunoreactive neurons, and 48% of choline acetyltransferase (ChAT)-immunoreactive neurons. At 2, 4 and 8 weeks after spinal hemisection, CS-A–positive PNNs accounted for 8.4%–9.9% of HuC/D-immunoreactive neurons, and 50%–60% of ChAT-immunoreactive neurons, which was not significantly different from intact fish. Chondroitin sulfate C (CS-C)–positive PNNs accounted for 6.4% of HuC/D-immunoreactive neuron, and 67% of ChAT-immunoreactive neurons in intact fish. At 2, 4 and 8 weeks after spinal hemisection, CS-C–positive PNNs accounted for 7.9%, 5.5% and 4.3%, respectively, of HuC/D-immunoreactive neurons, and 65%, 52% and 42%, respectively, of ChAT-immunoreactive neurons, demonstrating a significant decrease at 4 and 8 weeks after spinal hemisection. Among ventral horn neurons that received descending axons labeled with tetramethylrhodamine dextran amine (RDA) applied at the level of the first spinal nerve, CS-A–positive PNNs accounted for 53% of HuC/D-immunoreactive neurons. At 2 and 4 weeks after spinal hemisection, CS-A–positive PNNs accounted for 57% and 56% of HuC/D-immunoreactive neurons, which was not significantly different from intact fish. CS-C–positive PNNs, accounted for 48% of HuC/D-immunoreactive neurons that received RDA-labeled axons. At 2 and 4 weeks after spinal hemisection, CS-C–positive PNNs significantly decreased to 22% of the HuC/D-immunoreactive neurons, and by 4 weeks after spinal hemisection they had returned to 47%. These findings suggest that CS expression is maintained in the PNNs after spinal cord lesion, and that the descending axons regenerate to preferentially terminate on neurons not covered with CS-C–positive PNNs. Therefore, CS-C in the PNNs possibly inhibits new contact with descending axons, and plasticity in the spinal neurons might be endowed by downregulation of CS-C in the PNNs in the regeneration process after spinal hemisection in goldfish.

## Introduction

Perineuronal nets (PNNs) are reticular structures that surround the cell bodies and proximal dendrites of various neurons in the central nervous system. PNNs comprise extracellular matrix molecules such as hyaluronan, chondroitin sulfate proteoglycans (CSPGs), tenascin-R, and link proteins that interact with both hyaluronan and CSPGs (Kwok et al., 2011). PNNs are formed relatively late in the developmental process as mature synaptic connections are established. A role for PNNs is implicated in neuroprotection, ionic buffering and synaptic stabilization and maturation, i.e., restricting the formation of new contacts with advancing axons (Berardi et al., 2004; Morawski et al., 2004; Dityatev et al., 2007; Karetko and Skangiel-Kramska, 2009; Dzyubenko et al., 2016). The formation of PNNs is considered to coincide with the closure of the critical period for plasticity (Wang and Fawcett, 2012). PNNs in the rat spinal cord are formed in the second postnatal week. In PNNs, one CSPG type, aggrecan, is observed at postnatal day 7 (P7), although other CSPGs are not observed until P14 or P21, and persist into adulthood (Galtrey et al., 2008).

The mechanisms by which PNNs affect neuronal plasticity remain to be elucidated. One possible mechanism is that CSPGs in the PNNs directly inhibit neurite outgrowth to restrict new neuronal contacts. CSPGs are a proteoglycan subset containing core proteins and glycosaminoglycan side-chains of chondroitin sulfate (CS-GAG) that covalently bind to core proteins. Inhibition of axonal regeneration is largely due to CS-GAGs (Dou and Levine, 1995; Bradbury et al., 2002; Laabs et al., 2007). CS-GAGs are composed of repeating chondroitin sulfate (CS) disaccharide units formed by N-acetyl galactosamine (GalNAc) and glucuronic acid (GlcA), and modified by regional sulfation. CS disaccharides monosulfated in the 4 or 6 position of the GalNAc residue are referred to as chondroitin sulfate A (CS-A) or Chondroitin sulfate C (CS-C), respectively. In addition, CS disaccharides disulfated at the 2 and 6 positions of the GlcA and GalNAc (CS-D), respectively, and those disulfated at the 4 and 6 positions of GalNAc (CS-E) are reported (Sugahara et al., 2003). Some studies suggest that the effects of CSs on axonal outgrowth differ according to the sulfation pattern (Dou and Levine, 1995; Wang et al., 2008; Lin et al., 2011; Brown et al., 2012; Swarup et al., 2013).

The expression of CSPGs changes dramatically after central nervous system injury. After spinal cord injury, there is a significant increase in some types of CSPGs in the extracellular matrix surrounding the lesion site and a decrease in other types (Jones et al., 2003; Tang et al., 2003). CSPGs surrounding the lesion site are thought to be major growth-inhibitory factors, forming a chemical barrier that prevents the growth of regenerating axons (Snow et al., 1990; McKeon et al., 1991, 1995; Silver and Miller, 2004). A bacterial enzyme chondroitinase ABC (ChABC), degrades GAG side-chains from CSPG core proteins, rendering the CSPGs less inhibitory to axonal growth. Many reports show enhanced growth and regeneration of axons after *in vivo* ChABC application in several injury models (Moon et al., 2001; Bradbury et al., 2002; reviewed in Bradbury and Carter, 2011). Following spinal injury in the rat, ChABC treatment alone promotes the regrowth of spinal projections such as corticospinal projections, sensory dorsal column projections, and spinocerebellar projections beyond the lesion site (Bradbury et al., 2002; Yick et al., 2003; Barritt et al., 2006; Huang et al., 2006; Novotna et al., 2011). Removal of PNNs by the application of ChABC is also a potential therapeutic strategy to enhance neuronal plasticity and promote reorganization of connections in the spinal cord (Galtrey et al., 2007), but the expression of CSPGs in PNNs after injury has not been investigated in sufficient detail to confirm this notion.

The spinal cord injury model of the goldfish* Carassius auratus*, is unique in that the regenerating axons from the supraspinal origins grow past the lesion site to connect with spinal neurons below the lesion site (Takeda et al., 2007). In the present study, therefore, we examined CSPG expression in the PNNs after spinal hemisection in the goldfish. We used immunohistochemistry with specific monoclonal antibodies to examine two CS variants with different sulfation patterns, CS-A and CS-C. Both CS-A and CS-C negatively affect axonal growth in mammals (Wang et al., 2008; Swarup et al., 2013). Also, to evaluate whether CS-A and CS-C are co-expressed in the same PNN, we performed multiple labeling studies with tenascin-R, a pan-marker for CSPG, and CS-A or CS-C in the PNNs of intact fish.

## Materials and Methods

### Materials

Goldfish, *C. auratus* (*n* = 24; body weight 20–30 g), were obtained commercially (Nomoto Fish Farm Co. Ltd., Yokohama, Japan) and maintained in an aquarium at 25–27°C. This study was carried out in accordance with the recommendations of The Yokohama City University Committee for Animal Research. The protocol was approved by The Yokohama City University Committee for Animal Research. All procedures were performed according to the standards established by the NIH Guide for the Care and Use of Laboratory Animals and the Policies on the Use of Animals and Humans in Research. All efforts were made to minimize the number of animals used and their suffering.

### Spinal Cord Hemisection

**Figure 1:**
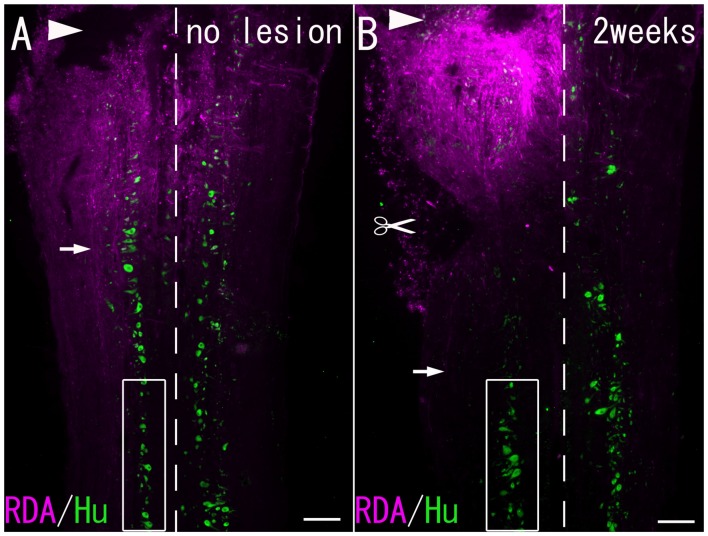
**(A)** A Horizontal section of the spinal cord without hemisection. **(B)** A Horizontal section of the spinal cord 2 weeks after hemisection. RDA-labeled fibers (arrows) were observed at the level caudal to the lesion site. Arrowheads: site of RDA application. Scissor mark: hemisection site. Dashed line: midline. Boxed area: the spinal cord under observation. Scale bar = 200 μm.

For better quantitative evaluation of the regenerating axons and behavioral activities, a lateral hemisection was made at the level between the first spinal nerve and the second spinal nerve (Figure 1). Full transection at the upper spinal level frequently produces permanent separation of the spinal cord. Lateral hemisection is a useful model for sequential observation of the injured tissue because of its efficient repair (Takeda et al., 2007, 2015). The fish (*n* = 18) were deeply anesthetized with 0.02% tricaine methanesulfonate (MS-222, Sigma-Aldrich Chemical Co., St. Louis, MO, USA) in water and placed on ice. The dorsal skin was incised at the level just caudal to the cranium, the muscles retracted, and the post-temporal bone and vertebrae exposed. The rostral segments of the spinal cord were exposed after removing the bones, and a frontal hemisection of the left side of the spinal cord was performed 500 μm caudal to the first spinal nerve. The hemisection was performed by inserting the blades of small scissors at a right angle to the spinal surface along the posterior median septum. After the wound was sutured and sealed with an aerosol plastic dressing (Yoshitomi-Seiyaku, Osaka, Japan), the fish were allowed to recover.

### Anterograde Tract-Tracing Study

Three intact fish and the lesioned fish at 2 weeks (*n* = 3) and 4 weeks (*n* = 3) after hemisection were deeply anesthetized with 0.02% MS-222 (Sigma-Aldrich Chemical Co.) in water and placed on ice. An incision was made in the appropriate region of the dorsal skin, and the rostral segments of the spinal cord were exposed. The spinal cord on the left side was cut at the level of the first spinal nerve. A piece of paper soaked with 10-mg/ml tetramethylrhodamine dextran amine (RDA; 1%; 0.5 μl; molecular weight = 3000; Invitrogen, Carlsbad, CA, USA) in phosphate-buffered saline (PBS; 0.1 M; pH 7.4) was inserted into the cut (Figure 1). The survival time after tracer application in the RDA group was 3 days.

### Tissue Preparation

Three intact fish; three RDA-administrated intact fish; 12 lesioned fish with survival periods of 1 week (*n* = 3), 2 weeks (*n* = 3), 4 weeks (*n* = 3), and 8 weeks (*n* = 3) after hemisection; and six RDA-administrated lesioned fish with survival periods of 2 weeks (*n* = 3) and 4 weeks (*n* = 3) after hemisection were anesthetized and perfused transcardially with saline containing 1% heparin, followed by 0.1 M phosphate buffer (PB, pH 7.4) containing 4% paraformaldehyde (PFA). The spinal cord, including the hemisectioned level, was removed immediately, postfixed in 0.1 M PB containing 4% PFA for 5–6 h at 4°C, and left overnight in 0.1 M PB containing 25% sucrose at 4°C for cryoprotection.

The spinal cord was embedded in Tissue-Tek OCT compound (Sakura, Tokyo, Japan) and frozen in liquid nitrogen. The frozen specimens were cut serially into 20-μm thick horizontal sections, and thaw-mounted on gelatin-coated slides in a cryostat (Moriyasu-Konetsu, Osaka, Japan) equipped with a microtome (Microm, Walldorf, Germany). Sections were arranged in five series comprising every fifth section. All the sections were dried for 1 h at room temperature, postfixed in 0.1 M PB containing 4% PFA for 30 min, and rinsed in 0.1 M PBS (pH 7.4) containing 0.3% Triton X-100 (PBST, pH 7.4) for 10–20 min.

### Fluorescence Immunohistochemistry

A series of spinal cord sections were incubated in a moist chamber overnight at 4°C with either: (1) a mixture of mouse monoclonal anti-HuC/D (5 μg/ml, Invitrogen, Carlsbad, CA, USA), rabbit polyclonal antibody against glial fibrillary acidic protein (GFAP; 15 μg/ml, Dako, Agilent Technologies, Glostrup, Denmark), and mouse monoclonal IgM antibody against CS-A (10 μg/ml, Clone 2H6, Cosmo Bio, Tokyo, Japan); (2) a mixture of mouse monoclonal anti-HuC/D (20 μg/ml, 16A11, Molecular Probes-ThermoFisher Scientific, Waltham, MA, USA), rabbit polyclonal antibody against GFAP (15 μg/ml, Dako), and mouse monoclonal IgM antibody against CS-C (1:20, Clone 3B3, Cosmo Bio); (3) a mixture of goat polyclonal antibody against choline acetyltransferase (ChAT; 1:100, AB144P, Merck KGaA, Darmstadt, Germany) and mouse monoclonal IgM antibody against CS-A (10 μg/ml, Clone 2H6, Cosmo Bio); (4) a mixture of goat polyclonal antibody against ChAT (1:100, AB144P, Merck KGaA) and mouse monoclonal IgM antibody against CS-C (10 μg/ml, Clone 2H6, Cosmo Bio); (5) a mixture of goat polyclonal antibody against ChAT (1:100, AB144P, Merck KGaA), mouse monoclonal IgM antibody against CS-C (10 μg/ml, Clone 2H6, Cosmo Bio), and mouse monoclonal IgG antibody against tenascin-R (10 μg/ml, Synaptic Systems, Gottingen Germany); or (6) a mixture of goat polyclonal antibody against ChAT (1:100, AB144P, Merck KGaA), mouse monoclonal IgM antibody against CS-C (10 μg/ml, Clone 2H6, Cosmo Bio), and mouse monoclonal IgG antibody against tenascin-R (10 μg/ml, Synaptic Systems), diluted with 1% normal donkey serum, 0.2% bovine serum albumin, and 0.1% NaN_3_ in 0.1 M PBST. For CS-C staining, the sections were pre-incubated with ChABC (0.2 units/ml, Sigma-Aldrich) in 50 mM Tris-HCl (pH 8.0) containing 50 mM acetic acid at 37°C for 1 h.

After several rinses with 0.1 M PBST, the sections were incubated for 3 h at room temperature with a mixture of secondary antibodies, i.e., cyanine Cy3-conjugated donkey anti-mouse IgG (10 μg/ml; Jackson ImmunoResearch Laboratories, West Grove, PA, USA), Alexa Fluor 488-conjugated donkey anti-mouse IgG (10 μg/ml; Jackson ImmunoResearch Laboratories), cyanine Cy5-conjugated donkey anti-mouse IgM (10 μg/ml; Jackson ImmunoResearch Laboratories), Alexa Fluor 488-conjugated donkey anti-mouse IgM (10 μg/ml; Jackson ImmunoResearch Laboratories), cyanine Cy5-conjugated donkey anti-rabbit IgG (10 μg/ml; Jackson ImmunoResearch Laboratories), cyanine Cy3-conjugated donkey anti-goat IgG (10 μg/ml; Jackson ImmunoResearch Laboratories), and aminomethylcoumarin-conjugated donkey anti-goat IgG (10 μg/ml; Jackson ImmunoResearch Laboratories), diluted with 1% normal donkey serum, 0.2% bovine serum albumin, and 0.1% NaN_3_ in 0.1 M PBST. In some sections, cell nuclei were counterstained with 4’, 6-diamino-2-phenylindole solution (10 μg/ml; Dojindo Molecular Technologies, Kumamoto, Japan) at room temperature for 3 h.

The specificity of the antibodies was verified by incubation with 0.5% normal mouse serum (Jackson ImmunoResearch Laboratories) or 0.5% normal rat serum (Jackson ImmunoResearch Laboratories) instead of the primary antibodies.

### Observation and Imaging

All the sections were examined with an epifluorescence microscope (Leica DMR; Leica, Wetzlar, Germany) equipped with excitation filters. Images obtained with a CCD camera (Leica DC 20; Leica) were digitally transferred to a computer using DC Viewer Software (Leica). Some sections were also examined with confocal laser scanning microscopy (Zeiss LSM 510; Carl-Zeiss, Jena, Germany) to construct three-dimensional images. Contrast and brightness were adjusted with Adobe Photoshop Software (Adobe, San Jose, CA, USA). The spinal neurons in the ventral horn at the level of the second spinal nerve, corresponding to the level 1–2 mm caudal to the hemisection, were analyzed (Figure 1).

We observed the immunoreactivity for CSs around the cell bodies of HuC/D-immunoreactive neurons and ChAT-immunoreactive neurons, and identified them as CS-positive PNNs when more than half of the outer circumference of the cell was covered with nets in which CS-immunoreactivity was stronger than the surrounding tissue. The assessment was independently performed by two investigators (A.K. and M.S.). Consensus between the two investigators was required to score them as positive.

### Statistical Analysis

The number of all HuC/D-immunoreactive neurons and ChAT-immunoreactive neurons was counted in fish without hemisection (*n* = 3), and fish at 1 week (*n* = 3), 2 weeks (*n* = 3), 4 weeks (*n* = 3) and 8 weeks (*n* = 3) after hemisection. The percentage of neurons covered with CS-A–positive PNNs and CS-C–positive PNNs among all HuC/D-immunoreactive neurons or all ChAT-immunoreactive neurons was calculated in the spinal cord on the injured side.

The number of HuC/D-immunoreactive neurons in contact with RDA-labeled terminals in the ventral horn was counted in fish without hemisection (*n* = 3), and in fish at 2 (*n* = 3) and 4 (*n* = 3) weeks after hemisection. The percentage of neurons covered with CS-A–positive PNNs and CS-C–positive PNNs among all HuC/D-immunoreactive neurons was calculated. Statistical analysis was performed using a one-tailed Mann-Whitney *U*-test. A *p*-value of less than 0.05 was considered significant.

## Results

### PNNs Positive for CS Expression

**Figure 2:**
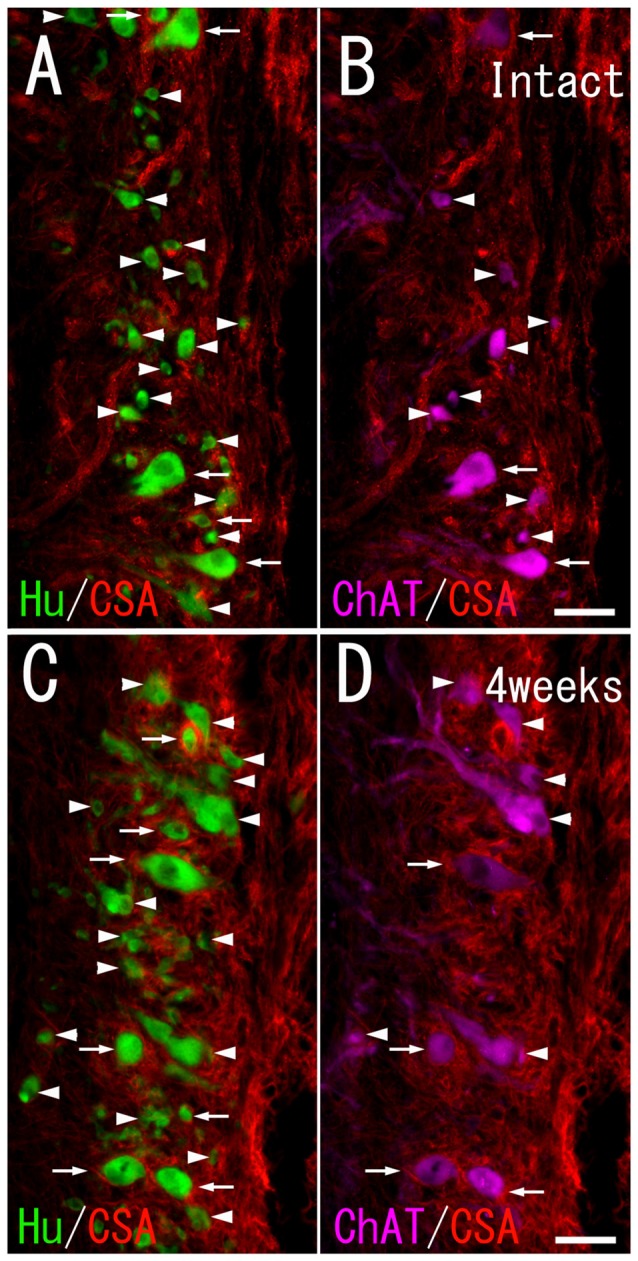
Chondroitin sulfate A (CS-A) immunoreactivity on HuC/D-immunoreactive neurons and ChAT-immunoreactive neurons in the ventral horn of intact fish **(A,B)**, and fish 4 weeks after spinal hemisection **(C,D)**. **(A,C)** Perineuronal nets (PNNs) positive for CS-A (red; indicated by arrows), and negative for CS-A (indicated by arrowheads) were observed on neurons positive for HuC/D (green) at the level caudal to the hemisection. **(B,D)** PNNs positive for CS-A (red; indicated by arrows), and negative for CS-A (indicated by arrowheads) were also observed on neurons positive for ChAT (purple) at the level caudal to the hemisection. Dashed line: midline. Scale bars = 50 μm.

**Figure 3:**
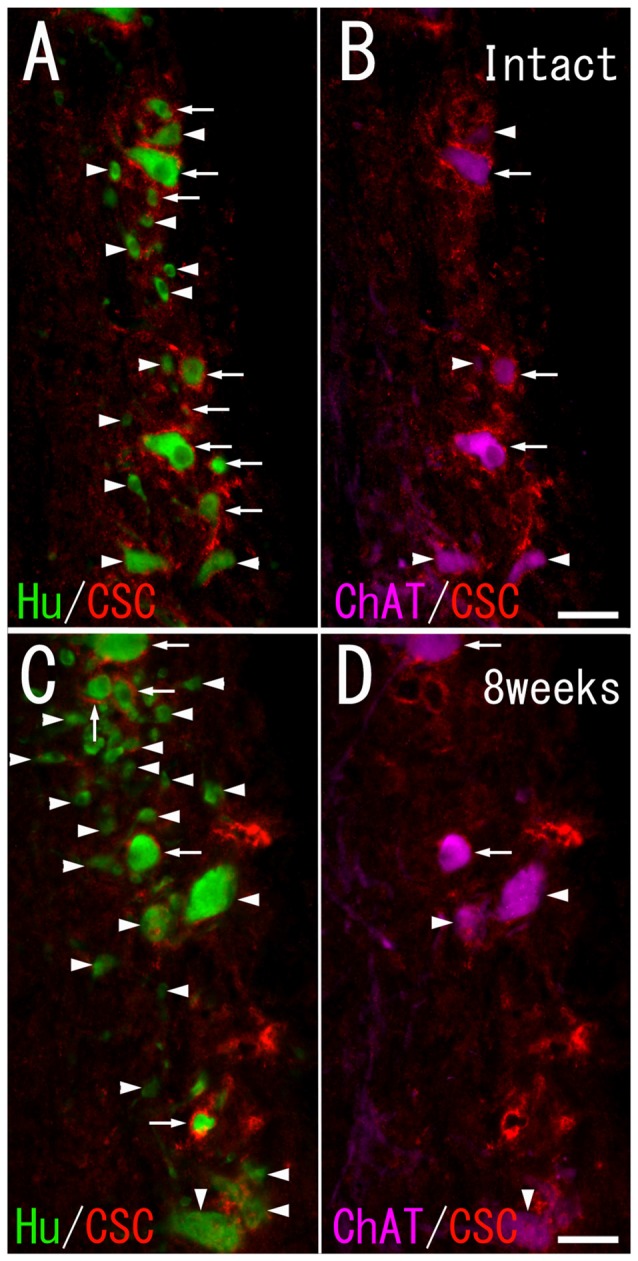
CS-C immunoreactivity on HuC/D-immunoreactive neurons and ChAT-immunoreactive neurons in the ventral horn of intact fish **(A,B)**, and fish 8 weeks after spinal hemisection **(C,D)**. **(A,C)** PNNs positive for CS-C (red; indicated by arrows), and negative for CS-C (indicated by arrowheads) were observed on neurons positive for HuC/D (green) at the level caudal to the hemisection. **(B,D)** PNNs positive for CS-C (red; indicated by arrows), and negative for CS-C (indicated by arrowheads) were also observed on neurons positive for ChAT (purple) at the level caudal to the hemisection. Dashed line: midline. Scale bars = 50 μm.

In the intact spinal cord, many CS-A–positive PNNs were present around HuC/D-immunoreactive neurons in the ventral horn. Most of the CS-A–positive PNNs were observed around large neurons, but some were also observed around small neurons (Figure 2A). In the HuC/D–CS-A double immunohistochemistry study performed in intact fish, the total number of HuC/D-immunoreactive neurons counted on the left side of the ventral horn was 1803. CS-A–positive PNNs accounted for 5.0% of all HuC/D-immunoreactive neurons in the ventral horn. CS-C–positive PNNs were also observed around HuC/D-immunoreactive neurons in the ventral horn (Figure 3A). In the HuC/D–CS-C double immunohistochemistry study performed in intact fish, the total number of HuC/D-immunoreactive neurons counted on the left side of the ventral horn was 2511. CS-C–positive PNNs accounted for 6.4% of all HuC/D-immunoreactive neurons.

CS-A–positive PNNs on HuC/D-immunoreactive neurons were observed after spinal hemisection (Figure 2C). In the HuC/D–CS-A double immunohistochemistry study performed 1, 2, 4 and 8 weeks after spinal hemisection, the total number of HuC/D-immunoreactive neurons counted on the injured side of the ventral horn was 1903, 2381, 2171 and 1559, respectively. The percentage of neurons covered with CS-A–positive PNNs among all HuC/D–positive neurons on the injured side of the ventral horn at 1, 2, 4 and 8 weeks after spinal hemisection was 7.8%, 9.9%, 8.8% and 8.4%, respectively. CS-C–positive PNNs on HuC/D-immunoreactive neurons were also observed after spinal hemisection (Figure 3C). In the HuC/D–CS-C double immunohistochemistry study performed 1, 2, 4, and 8 weeks after spinal hemisection, the total number of HuC/D-immunoreactive neurons counted on the injured side of the ventral horn was 2095, 2547, 2750 and 2092, respectively. The percentage of neurons covered with CS-C–positive PNNs among all HuC/D-immunoreactive neurons on the injured side of the ventral horn at 1, 2, 4 and 8 weeks after spinal hemisection was 6.0%, 7.9%, 5.5% and 4.3%, respectively.

### PNN Expression on ChAT-Immunoreactive Neurons

CS-A–positive PNNs on ChAT-immunoreactive neurons in the ventral horn were observed in intact fish (Figure 2B). In the ChAT–CS-A double immunohistochemistry study performed in intact fish, the total number of ChAT-immunoreactive neurons counted on the left side of the ventral horn was 246. The percentage of neurons covered with CS-A–positive PNNs among ChAT-positive neurons was 48%. Many ChAT-positive neurons covered with CS-A–positive PNNs were positive for tenascin-R. The percentage of neurons covered with CS-A–positive PNNs among ChAT-positive neurons with PNNs positive for tenascin-R was 68%. CS-C–positive PNNs on ChAT-immunoreactive neurons in the ventral horn were also observed in intact fish (Figure 3B). In the ChAT–CS-C double immunohistochemistry study, the total number of ChAT-immunoreactive neurons counted on the left side of the ventral horn in intact fish was 241. The percentage of neurons covered with CS-C–positive PNNs among ChAT-positive neurons was 67%. Most ChAT-positive neurons covered with CS-C–positive PNNs were positive for tenascin-R. The percentage of neurons covered with CS-C–positive PNNs among ChAT-positive neurons with PNNs positive for tenascin-R was 96%.

**Figure 4:**
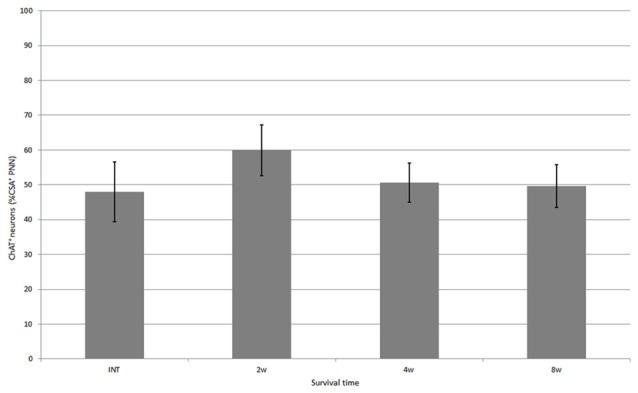
Percentage of neurons covered with CS-A–immunoreactive PNNs among all ChAT-positive neurons in the ventral horn in intact fish, and fish at 2, 4 and 8 weeks after spinal hemisection. Values are means ± SE.

**Figure 5:**
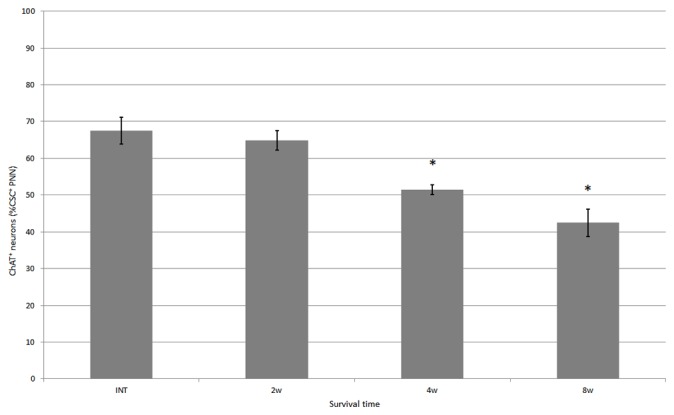
Percentage of neurons covered with CS-C–immunoreactive PNNs among all ChAT-positive neurons in the ventral horn in intact fish, and fish at 2, 4 and 8 weeks after hemisection, respectively. Values are means ± SE. Significant difference compared with intact fish is indicated by * (*p* < 0.05).

CS-A–positive PNNs on ChAT-immunoreactive neurons in the ventral horn were also observed in fish after spinal hemisection (Figure 2D). In the ChAT–CS-A double immunohistochemistry study performed 2, 4 and 8 weeks after spinal hemisection, the total number of ChAT-immunoreactive neurons counted on the injured side of the ventral horn was 340, 228 and 183, respectively. The percentage of neurons covered with CS-A–positive PNNs among ChAT-positive neurons at 2, 4 and 8 weeks after spinal hemisection was 60%, 51% and 50%, respectively, and was not significantly different among time-points or between intact and hemisectioned fish (Figure 4). The intensity of the CS-A immunoreactivity was not significantly different among time-points or between intact and hemisectioned fish. CS-C–positive PNNs on ChAT-immunoreactive neurons in the ventral horn were also observed in fish after spinal hemisection (Figure 3D). In the ChAT–CS-C double immunohistochemistry study performed 2, 4 and 8 weeks after spinal hemisection, the total number of ChAT-immunoreactive neurons counted on the injured side of the ventral horn was 392, 235 and 200, respectively. The percentage of neurons covered with CS-C–positive PNNs among ChAT-positive neurons at 2, 4 and 8 weeks after spinal hemisection was 65%, 52% and 42%, respectively, and was significantly decreased at 4 and 8 weeks compared with 2 weeks after spinal hemisection (Figure 5). The intensity of the CS-C immunoreactivity was not significantly different among time-points or between intact and hemisectioned fish.

### PNN Expression on Neurons in Contact With RDA-Labeled Terminals

In RDA-administrated fish without hemisection, some HuC/D-immunoreactive neurons in contact with RDA-labeled terminals were observed in the ventral horn on the side ipsilateral to the RDA application, but not on the contralateral side. The HuC/D-immunoreactive neurons with RDA-labeled terminals were frequently covered with PNNs positive for CS-A. In the RDA/HuCD–CS-A double immunohistochemistry study performed in intact fish, the total number of neurons with RDA-labeled terminals counted on the left side of the ventral horn was 248. Among all HuC/D-immunoreactive neurons in contact with RDA-labeled terminals, 53% of neurons were covered with CS-A–positive PNNs. The HuC/D-immunoreactive neurons with RDA-labeled terminals were also frequently covered with PNNs positive for CS-C. In the RDA/HuCD–CS-C double immunohistochemistry study performed in intact fish, the total number of neurons with RDA-labeled terminals counted on the left side of the ventral horn was 136. Among all HuC/D-immunoreactive neurons in contact with RDA-labeled terminals, 48% of neurons were covered with CS-C–positive PNNs.

**Figure 6:**
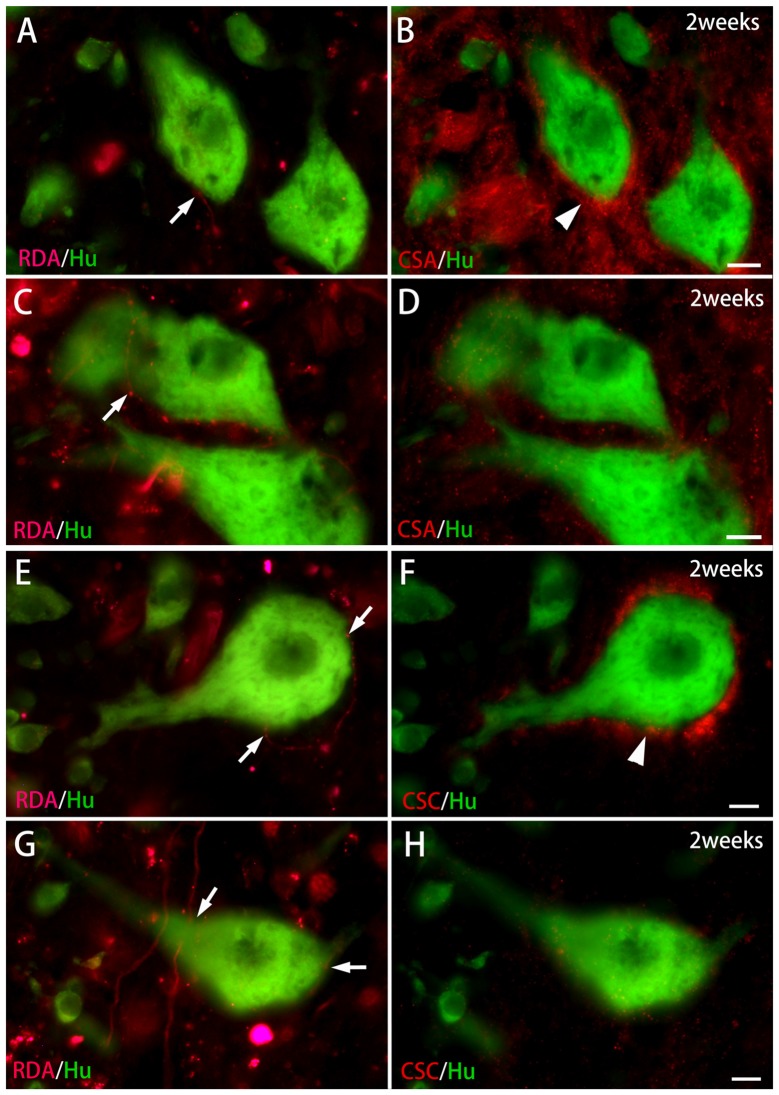
CS immunoreactivity on HuC/D-immunoreactive neurons in contact with RDA-labeled terminals in the ventral horn 2 weeks after spinal hemisection. **(A,B)** RDA-labeled axon terminals (indicated by an arrow in **A**) were in contact with HuC/D-immunoreactive neurons (green) at the level caudal to the hemisection. PNNs positive for CS-A (red; indicated by an arrowhead in** B**) were observed on the same HuC/D-immunoreactive neurons.** (C,D)** RDA-labeled axon terminals (indicated by an arrow in **C**) were in contact with HuC/D-immunoreactive neurons (green). No PNNs positive for CS-A were observed on the same HuC/D-immunoreactive neurons in **(D)**. **(E,F)** RDA-labeled axon terminals (indicated by arrows in **E**) were in contact with a HuC/D-immunoreactive neuron (green) at the level caudal to the hemisection. PNNs positive for CS-C (red; indicated by an arrowhead in** F**) were observed on the same HuC/D-immunoreactive neuron. **(G,H)** RDA-labeled axon terminals (indicated by arrows in **G**) were in contact with HuC/D-immunoreactive neurons (green). No PNNs positive for CS-C were observed on the same HuC/D-immunoreactive neurons in **(H)**. Dashed line: midline. Scale bars = 10 μm.

**Figure 7:**
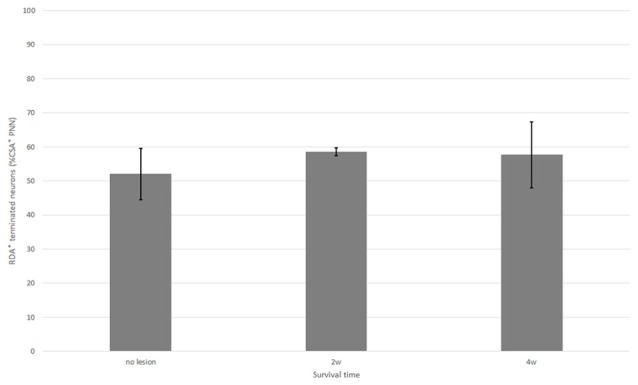
Percentage of neurons with CS-A–positive PNNs among all HuC/D-immunoreactive neurons in contact with RDA-labeled terminals in the ventral horn in fish with no hemisection, and fish at 2 and 4 weeks after hemisection. Values are means ± SE.

**Figure 8:**
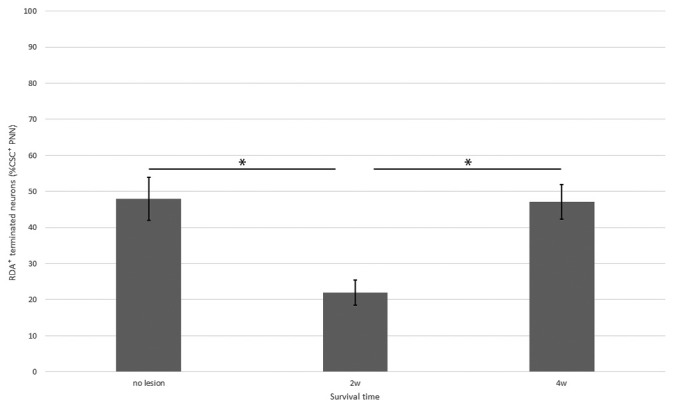
Percentage of neurons with CS-C–positive PNNs among all HuC/D-immunoreactive neurons in contact with RDA-labeled terminals in the ventral horn in fish with no hemisection, and fish at 2 and 4 weeks after hemisection. Values are means ± SE. Significant differences are indicated by * (*P* < 0.05).

In the spinal cord 2 weeks after hemisection, HuC/D-immunoreactive neurons in contact with RDA-labeled terminals were also observed in the ventral horn on the side ipsilateral to the RDA application. CS-A–positive PNNs were observed around the HuC/D-immunoreactive neurons in contact with RDA-labeled terminals (Figure 6). In the RDA/HuCD–CS-A double immunohistochemistry study performed 2 weeks after spinal hemisection, the total number of neurons with RDA-labeled terminals counted on the injured side of the ventral horn was 138. The percentage of neurons covered with CS-A–positive PNNs among all HuC/D-immunoreactive neurons in contact with RDA-labeled terminals was 57%, and not significantly altered in the ventral horn 2 weeks after hemisection (Figure 7). CS-C–positive PNNs were also observed around the HuC/D-immunoreactive neurons in contact with RDA-labeled terminals. In the RDA/HuC/D–CS-C double immunohistochemistry study performed 2 weeks after spinal hemisection, the total number of neurons with RDA-labeled terminals counted on the injured side of the ventral horn was 80. The percentage of neurons covered with CS-C–positive PNNs among all HuC/D-immunoreactive neurons in contact with RDA-labeled terminals was 22%, which was significantly lower than that in fish with no hemisection (Figure 8).

HuC/D-immunoreactive neurons in contact with RDA-labeled terminals were also observed on the side ipsilateral to the RDA application at 4 weeks after hemisection. CS-A–positive PNNs were observed around the HuC/D-immunoreactive neurons in contact with RDA-labeled terminals. In the RDA/HuC/D–CS-A double immunohistochemistry study performed 4 weeks after spinal hemisection, the total number of neurons with RDA-labeled terminals counted on the injured side of the ventral horn was 278. The percentage of neurons covered with CS-A–positive PNNs among all HuC/D-immunoreactive neurons in contact with RDA-labeled terminals was 56%, which was not significantly altered in the ventral horn 4 weeks after hemisection (Figure 7). CS-C–positive PNNs were also observed around the HuC/D-immunoreactive neurons in contact with RDA-labeled terminals. In the RDA/HuC/D–CS-C double immunohistochemistry study performed at 4 weeks after spinal hemisection, the total number of neurons with RDA-labeled terminals counted on the injured side of the ventral horn was 245. The percentage of neurons covered with CS-A–positive PNNs among all HuC/D-immunoreactive neurons in contact with RDA-labeled terminals was 47%, which was significantly increased to the level in fish with no hemisection (Figure 8).

## Discussion

### PNN Expression in the Goldfish Spinal Cord

The presence of CS immunoreactivity on the surface of many Hu-immunoreactive cells in intact fish suggests that PNNs are formed and maintained in the adult stage in goldfish. The present study revealed that more than half of the ChAT-positive neurons were positive for CS, although less than 10% of all Hu-positive spinal neurons were CS-positive. Therefore, PNNs preferentially form around the motor neurons in the goldfish spinal cord, whereas interneurons might be less well covered with PNNs. Following spinal hemisection, CS expression was also observed in PNNs surrounding Hu-immunoreactive neurons or ChAT-immunoreactive neurons at all postoperative time-points examined. Thus, PNNs might have beneficial effects on spinal neurons, protecting them from various cytotoxic substances produced by the damaged tissue.

### Differential Expression of CS-A and CS-C in the PNNs

CS-A and CS-C are disaccharide units that form the CS-GAG chains to bind core proteins of CSPGs. In the present study, we used monoclonal antibody clone 2H6 to detect CS-A. This antibody effectively recognizes CS-A, especially in the central nervous system (Oohira et al., 1994). We used monoclonal antibody clone 3B3 to detect CS-C. This antibody was raised against CS-C neoepitopes on CS-GAG chains that were pre-digested with either ChABC or chondroitinase ACII, and efficiently recognizes CS-C stubs on the CS-GAG chains (Caterson, 2012). Therefore, before immunostaining using this antibody, we pretreated the tissue with ChABC.

The present results showed that the percentage of neurons with CS-A–positive PNNs among all spinal neurons was not significantly changed after spinal hemisection, whereas the percentage of neurons with CS-C–positive PNNs among all spinal neurons tended to decrease. Similar observations were obtained for PNNs surrounding the ChAT-positive neurons. The present multiple immunofluorescence study in intact fish showed that the percentage of neurons covered with CS-A–positive PNNs among ChAT-positive neurons with PNNs positive for tenascin-R was 68%, whereas the percentage of neurons covered with CS-C–positive PNNs among ChAT-positive neurons with PNNs positive for tenascin-R was 96%. It is certain, therefore, that both CS-A and CS-C are frequently expressed in the same PNNs surrounding ChAT-positive neurons. It is also possible that both CS-A units and CS-C units were contained in the same chain. Nevertheless, the present study highlights the relative change in the proportion of CS-A and CS-C in PNNs. Thus, the percentage of neurons with CS-A–positive PNNs was not significantly different, whereas the percentage of neurons with CS-C–positive PNNs gradually decreased after spinal hemisection. These results suggest that the expression of CS-A and CS-C might be differentially regulated in the PNNs.

### Relationship Between PNN Expression and Re-Innervation of Descending Axons

In goldfish, spinal hemisection produces scar tissue with dense collagen fibers at the lesion site. Descending spinal projections, however, spontaneously regenerate across the scar to re-innervate the spinal neurons below the lesion site. When RDA is injected into the nucleus of the medial longitudinal fasciculus in the midbrain, the number of anterogradely-labeled terminal boutons or varicosities in close apposition to spinal neurons is significantly decreased 4 days after spinal transection, but returns to the control level within 6 weeks after spinal transection (Takeda et al., 2007). Together with previous results showing that the number of descending projections beyond the lesion site increases between 1 and 4 weeks after spinal hemisection (Takeda et al., 2015), the present data suggest that new synaptic contacts between ventral horn neurons and descending fibers formed within 4 weeks after spinal hemisection.

The findings of the present study demonstrated that the percentage of neurons covered with PNNs positive for CS-C, but not CS-A, among all spinal neurons in contact with RDA-labeled terminals was decreased 2 weeks after hemisection. Thus, the descending axons that regenerated beyond the scar tissue formed terminal buttons preferentially on neurons not covered with PNNs positive for CS-C. These findings suggest that CS-C in the PNNs possibly inhibits the formation of new contacts with the descending axons, and that CS-A in the PNNs, on the other hand, is unlikely to play such an inhibitory role. The plasticity in the spinal neurons might be endowed by the downregulation of CS-C in the PNNs in the regeneration process after spinal injury. Furthermore, the increase in the percentage of neurons covered with PNNs positive for CS-C among all spinal neurons in contact with RDA-labeled terminals at 4 weeks after spinal hemisection suggests that the critical period for plasticity closes within 4 weeks after hemisection. These results appear to be in contrast with previous findings that neuronal plasticity persists when CS-C is upregulated in the neocortex (Miyata et al., 2012). Therefore, the relationship between CS sulfation patterns and neuronal plasticity might be quite complex and differ depending on cell type.

### Relationship Between PNN Expression and Functional Improvement

The present study showed that 48% and 67% of intact motor neurons were covered by PNNs positive for CS-A and CS-C, respectively. The percentage of motor neurons with CS-positive PNNs in goldfish, therefore, might be higher than that in rats, in which less than 30% of motor neurons are positive for *Wisteria floribunda* agglutinin staining (Galtrey et al., 2008). In the present study, we did not examine the expression of CSs on ChAT-immunoreactive neurons in contact with RDA-labeled terminals after spinal hemisection. Therefore, we could not evaluate the relationship between CSs in PNNs on motor neurons and contact formation with the regenerating axons following spinal injury. Our previous study, on the other hand, showed that some regenerating axons of the nucleus of the medial longitudinal fasciculus formed varicose terminals directly on motor neurons after spinal hemisection (Takeda et al., 2007). Therefore, changes in the expression of CSs in PNNs on motor neurons may induce contacts between the descending projections and motor neurons, resulting in functional motor recovery in fish.

In mammals, spinal cord injury induces a significant upregulation of CSPGs in the PNNs on motor neurons 1 week after spinal injury (Alilain et al., 2012). This finding is in contrast to the present results in goldfish, in which no significant changes in immunoreactivity for CS-A or CS-C were observed 1–2 weeks after spinal hemisection. The differential expression of CSPGs in response to spinal cord injury might be related to the success of motor function recovery in fish.

## Author Contributions

KF and AT designed the study; AT and MS conducted experiments; AT analyzed the results; KF wrote the manuscript.

## Conflict of Interest Statement

The authors declare that the research was conducted in the absence of any commercial or financial relationships that could be construed as a potential conflict of interest. The reviewer OAT-C and handling Editor declared their shared affiliation.
